# Immunopathogenesis in Myasthenia Gravis and Neuromyelitis Optica

**DOI:** 10.3389/fimmu.2017.01785

**Published:** 2017-12-12

**Authors:** Zhen Wang, Yaping Yan

**Affiliations:** ^1^Key Laboratory of the Ministry of Education for Medicinal Resources and Natural Pharmaceutical Chemistry, National Engineering Laboratory for Resource Development of Endangered Crude Drugs in Northwest China, College of Life Sciences, Shaanxi Normal University, Xi’an, China; ^2^Tianjin Medical University General Hospital, Tianjin Neurological Institute, Tianjin, China

**Keywords:** neuromyelitis optica spectrum disorders, myasthenia gravis, channelopathy, humoral immunity, inflammation

## Abstract

Myasthenia gravis (MG) and neuromyelitis optica (NMO) are autoimmune channelopathies of the peripheral neuromuscular junction (NMJ) and central nervous system (CNS) that are mainly mediated by humoral immunity against the acetylcholine receptor (AChR) and aquaporin-4 (AQP4), respectively. The diseases share some common features, including genetic predispositions, environmental factors, the breakdown of tolerance, the collaboration of T cells and B cells, imbalances in T helper 1 (Th1)/Th2/Th17/regulatory T cells, aberrant cytokine and antibody secretion, and complement system activation. However, some aspects of the immune mechanisms are unique. Both targets (AChR and AQP4) are expressed in the periphery and CNS, but MG mainly affects the NMJ in the periphery outside of CNS, whereas NMO preferentially involves the CNS. Inflammatory cells, including B cells and macrophages, often infiltrate the thymus but not the target—muscle in MG, whereas the infiltration of inflammatory cells, mainly polymorphonuclear leukocytes and macrophages, in NMO, is always observed in the target organ—the spinal cord. A review of the common and discrepant characteristics of these two autoimmune channelopathies may expand our understanding of the pathogenic mechanism of both disorders and assist in the development of proper treatments in the future.

## Introduction

Myasthenia gravis (MG) is an autoimmune disease in which antibodies target postsynaptic membrane components at the neuromuscular junction (NMJ) and is characterized by fluctuating muscle weakness and fatigue (1–3). MG involves specific skeletal muscles, frequently including ocular, bulbar, and proximal extremity muscles but also affects respiratory muscles in severe cases (4, 5). The disease begins with an acute or subacute onset, improves with spontaneous remission or treatment, and relapses after variable intervals (6, 7). As the most important biomarkers in diagnosis, antibodies comprise a series of immunoglobulins (Igs) binding to acetylcholine receptors (AChR)—an ion channel protein, muscle-specific kinase (MuSK), and lipoprotein receptor-related protein 4 (LRP4) or other postsynaptic proteins (4). Based on the antibody profile, clinical presentation, age of onset, and thymic pathology, patients can be divided into several subtypes: MG with anti-AChR antibodies (AChR-MG) of early-onset, late-onset or with thymoma; MG with anti-MuSK antibodies (MuSK-MG); MG with anti-LRP4 antibodies (LRP4-MG); ocular MG; and seronegative MG (1, 4). MG has a prevalence of 15–25 cases per 100,000 individuals and an annual incidence of 0.8–1 cases per 100,000 individuals (1, 8), and AChR-MG constitutes approximately 80% of all MG cases (4, 5). The age of onset and the female-to-male ratio varies between different subtypes (2, 4, 5). The disease is usually well controlled by immunosuppressive, symptomatic, supportive, or surgical treatment in most patients; however, only a few patients (22.2% of AChR-MG, 3.6% of MuSK-MG, and 21.9% of others) obtain full remission (1, 4, 9).

Neuromyelitis optica (NMO) is a severe, idiopathic, demyelinating disorder of the central nervous system (CNS) that has recently been recognized to be distinct from the classic demyelinating disease—multiple sclerosis (MS). NMO preferentially affects the optic nerve and spinal cord, but relatively spares the brain (10). With the discovery of the diagnostic biomarker—NMO-IgG (11), a better understanding of the pathogenesis of the disease was obtained and the clinical entity evolved. In 2015, the diagnostic criteria adopted the term neuromyelitis optica spectrum disorders (NMOSD) to incorporate inaugural or limited forms of NMO (idiopathic single or recurrent longitudinally extensive myelitis or recurrent or simultaneous bilateral optic neuritis), the involvement of the brain, coexistence with other autoimmune disorders, and Asian opticospinal MS (12). Most patients are seropositive for Ig G against aquaporin-4 (AQP4-IgG) (13–16), which is the most abundant water channel protein in astrocytes throughout the CNS (17, 18). Approximately 5–10% of patients are seropositive for antibodies against myelin oligodendrocyte glycoprotein (MOG-IgG) (19–21), and a few patients are dual-positive for both antibodies (22, 23). The prevalence and incidence of NMO/NMOSD are approximately 3.9–10 and 0.07–0.73 per 100,000, respectively, the median age of onset is 35–37 years and the female-to-male ratio is approximately 8–9:1 (24). Most patients have a relapsing course, with the interval between attacks ranging from months to years; the subsequent accumulation of disability leads to a poor prognosis despite the use of immunosuppressive treatment (10, 16, 25).

As autoimmune channelopathies in the periphery and CNS, MG and NMO share many similarities: (i) they develop based on a synergy between genetic factors and environmental effects (26, 27), (ii) the common female dominance in the prevalence of some major subtypes suggests an influence of gender on both diseases (28, 29), (iii) both depend on T cell-mediated, B cell-dependent immunopathology and the effects of antibodies and complements (30, 31), (iv) patients with the two disorders display similar relapsing courses and require chronic immunomodulatory management (1, 7, 10), (v) the disorders frequently coexist with other systemic or organ-specific autoimmune disorders (32, 33), and (vi) AChR-IgG and AQP4-IgG have been co-detected in patients with MG and NMOSD in a few studies (16, 33). MG and NMO may share similar pathogenic mechanisms; however, some discrepancies exist. AChR and AQP4 are expressed in the periphery and CNS (18, 34), whereas MG mainly affects the NMJ in the periphery outside of CNS (35), and NMO preferentially involves the CNS (36, 37). Inflammatory cells, including B cells and macrophages, often infiltrate the thymus but not the target—muscle in MG (38), whereas infiltration of inflammatory cells, mainly polymorphonuclear leukocytes and macrophages, in NMO, is always observed in the target organ—the spinal cord (39). A comparison of the pathogenesis, particularly the immune regulation, between MG and NMO is compelling, and will expand our understanding of the pathogenesis and assist in the future development of appropriate treatments.

## Inherited Susceptibility

The prevalence of familial and monozygotic patients has helped to explain the role of hereditary factors in pathogenesis. The frequency of familial MG in general patients is approximately 3–4% (40, 41), and the concordance between monozygotic MG twins is approximately 35% (42), both of these values are higher than the prevalence of 15–25/100,000 in the general population. Similarly, two studies reported a frequency of familial occurrence of NMOSD of approximately 3% (43, 44), which is greater than the prevalence of 0.52–4.4/100,000 in the general population. Based on these findings, genetic factors are likely to be involved in the susceptibility to both MG and NMOSD. However, the concordance of only 35% in monozygotic MG twins and rare reports of monozygotic NMOSD twins support the important role of environmental factors in the etiology (42, 45).

Human leukocyte antigen (HLA) genes are always strongly associated with many autoimmune diseases (46, 47). The AH8.1 haplotype has been reported to be linked to early-onset MG in a Caucasian population (48). Recently, a genome-wide association study in Turkey found that HLA-B*08:01 and HLA-C*07:01 are associated with early-onset AChR-MG; HLA-DQA1 and HLA-DQB1 are associated with late-onset AChR-MG; and HLA-DQB1*05:02 is associated with MuSK-MG (49). However, another North American and Italian study identified a link between HLA-DQA1 and both subtypes through different variants (50). According to two studies from China, the DQ9 haplotype and HLA-DRB1*09 alleles occur more frequently in a southern Han population with ocular MG and in northern Han patients with MG than in controls, respectively (51, 52). Several studies from different populations have together identified an association of DQ*5 alleles with MuSK-MG (53–56). In addition, some associated non-HLA loci have also been identified, such as cytotoxic T lymphocyte-associated protein 4, tumor necrosis factor receptor superfamily 11A (TNFRSF11A), zinc finger and BTB domain-containing 10 (ZBTB10), protein tyrosine phosphatase nonreceptor type 22 (PTPN22), tumor necrosis factor alpha-induced protein 3-interacting protein 1 (TNIP1), and receptor activator of nuclear factor κB ligand (50, 57, 58). Finally, the polymorphisms in CHRAN1 and CHRND encoding the subunits of AChR were found to confer an increased risk of MG (59, 60), suggesting that an aberrant AChR structure might contribute to autoimmunity.

An association between NMOSD and HLA has also been reported in different populations, although this notion was refuted in one study (61). DPB1*1501 has been reported to be associated with opticospinal forms of MS—a subgroup of NMOSD in Japan, despite its presence in 60% of the general population (62). DPB1*0501 was also shown to correlate with AQP4-IgG-positive NMO/NMOSD in southern Han Chinese and Japanese populations (63, 64). In a Spanish cohort, DRB1*03 was not only more frequent in patients with NMO than in healthy controls but was also associated with AQP4-IgG seropositivity (65). This allele was also confirmed in Afro-Caribbean, Brazilian, and south Indian patients with NMO (66–68). Similar to MG, some non-HLA loci are also likely related to NMO/NMOSD pathogenesis, such as the T cell receptor, cluster of differentiation 6, TNFRSF1A, CD58, interleukin (IL)-17A and IL-17F, and the CYP7A1 promoter (69–73). However, PTPN22, which is associated with MG and other autoimmune diseases, was not correlated with NMO (74). Unlike polymorphisms in the AChR gene in MG, polymorphisms in AQP4 are not associated with NMO susceptibility (75).

The obvious association between HLA and MG/NMO suggests that antigen-presenting cells (APCs) and lymphocytes might play important roles in transferring signals from the activated innate immune system into specific adaptive autoimmune responses and establishing long-lived memory autoimmunity. In addition, the polymorphisms in non-HLA genes involved in immune signaling might cumulatively contribute to the pathogenesis of MG and NMO by overcoming or lowering the thresholds for immune signaling.

## Epigenetic Mechanisms

Epigenetic mechanisms link the environmental factors and genetics in disease, which include microRNA, DNA methylation, and histone acetylation (38). Many aberrant microRNAs expression has been reported to be involved in MG, including miR-320a, miR-155, miR-146a, and let-7c in immune cells and miR-150 and miR-21 in sera (26, 38). Mamrut et al. recently studied the profile of transcriptome and methylome in MG and found extremely high similarity at the transcription and the DNA methylation levels not only between discordant twins but also between the healthy discordant twins and the whole MG patients group, which indicated the high importance of genetic predisposition in the pathogenesis of MG (76). In addition, many differentially expressed genes and methylated CpGs in peripheral monocytes were detected between MG patients and controls, which suggested numerous small changes at gene or methylation levels might together contribute to MG development (76). In NMO patients, a recent study found 17 microRNAs was upregulated and 25 microRNAs was downregulated compared with healthy controls (77). Interestingly, the downregulated expression of miR-150 and miR-21 in serum of NMO patients is different from the upregulated expression in whole blood of MG patients, which may be a result of different methods.

## Environmental Factors

Many environmental factors contribute to the onset and severity of autoimmune diseases as predisposing factors, such as diet, vitamin D, and microbiota, or lead to relapse as triggering factors, such as infections, pollutants, and pharmacological molecules (38).

Vitamin D deficiency has been found to be correlated with the prevalence of many autoimmune diseases, such as type I diabetes mellitus, MS, rheumatoid arthritis (RA), systemic lupus erythematosus (SLE), and inflammatory bowel diseases (78). Vitamin D contributes to the regulation of the immune system through multiple mechanisms, including regulation of the activation and differentiation of CD4 lymphocytes, the suppression of differentiation of monocytes into dendritic cells, the reduction of cytokine production, and stimulation of natural killer T cells (38). In patients with MG, vitamin D levels are decreased, and vitamin D improves the autoimmune response and fatigue (79). As shown in the study by Alahgholi-Hajibehzad et al., vitamin D significantly increases the function of regulatory T cells (Treg) derived from patients with MG *in vitro* (80), and complete remission of severe refractory MG was reported after treatment with a massive-dose of vitamin D; however, this finding remains to be confirmed by additional high-quality clinical trials (81). According to Mealy et al., vitamin D levels are significantly lower in patients with recurrent spinal cord disease, mainly including NMO/NMOSD (82); the finding was reproducible in other NMO/NMOSD studies (83–86). Among these studies, a group from south China found that vitamin D levels were inversely correlated with disease-related disability, clinical activity, and prognosis (83); however, Thai, Turkish, and Korean groups did not observe a correlation (84–86). Additional studies are needed to clarify whether low vitamin D levels are a predisposing factor for or a secondary consequence of NMO.

The gut microbiota consists of trillions of microorganisms that colonize the intestine and regulate the maturation and function of the host immune system (87). When the host changes his or her diet or lifestyle or overuses antibiotics, the susceptibility to autoimmune disorders may increase due to the altered symbiotic relationship between the host immune system and the microbiota (88). Despite considerable research on the relationship between the gut microbiota and other autoimmune diseases, studies of the microbiota in patients with MG are scarce. A mixture of probiotics was recently shown to reduce the clinical symptoms of experimental autoimmune MG by suppressing AChR-reactive lymphocytes and generating regulatory dendritic cells and Tregs (89). An investigation of the gut microbiota in patients with NMO revealed the overrepresentation of *Clostridium perfringens*, and the *C. perfringens* adenosine triphosphate-binding cassette transporter (ABC), shared a homologous sequence with AQP4 that could cross-react with T cells from patients with NMO (90, 91). This result provides a new cue for the pathogenesis of NMO, but further studies, including the establishment of appropriate animal models, are warranted.

Viral infections, particularly with Epstein–Barr virus (EBV), have been correlated with the pathogenesis of many autoimmune diseases in seroepidemiological and immunological studies (92). EBV-infected B cells have been detected in the target organs in many autoimmune diseases; similarly, these cells were also detected in the hyperplastic thymus of patients with MG (38, 93). High levels of antibodies against the type 1 nuclear antigen of EBV were recently shown to be more common in patients with MG (94). The virus might induce persistent inflammation in the thymus and initiate autoantigen sensitization, leading to the subsequent autoimmune response (92). However, this finding was not confirmed by two other studies (95). Antibodies against EBV were more frequently detected in the serum and cerebrospinal fluid (CSF) of patients with NMO than in controls, suggesting that EBV might be involved in NMO pathogenesis (96). In addition, a peptide derived from the TAX1BP1 protein of human T cell leukemia virus type 1 virus (HTLV-1), was used to immunize mice and induced the production of antibodies against the peptide and homologous AQP4 epitope without any brain lesions, suggesting that HTLV might also be implicated in the pathogenesis of NMO (97), although a previous clinical study argued against this view (98).

## Gender Bias

Most autoimmune diseases exhibit a higher incidence in females (99). Gonadal hormones and direct X-chromosome effects have been proposed to contribute to the sex bias (99). Compared with males, females have many differences in innate immunity and adaptive immunity (100). Females were revealed to have higher expression of some genes involved in toll-like receptor (TLR) pathways and stronger type I interferon (IFN) responses by transcriptional analyses (100, 101). In addition, females display higher phagocytic activities of neutrophils and macrophages, more efficient APCs and dysregulation of innate lymphoid cells (100, 102, 103). Females also have higher CD4^+^ T cell counts, higher CD4/CD8 ratios, higher basal Ig levels, and higher B cell numbers, as well as lower Treg counts (100, 104, 105). Moreover, peripheral blood mononuclear cells (PBMCs) produce more activated CD4^+^ T cells (100). Estrogens may play a major role in this effect as they can favor the follicular helper T cells (Tfh) response and affect B cell maturation, selection, and antibody secretion (29, 106). Furthermore, at specific doses, time points, and microenvironments, estrogens allow autoreactive B cells to escape from the normal tolerance mechanisms and to accumulate in sufficient numbers to induce autoimmunity (38, 107).

Obvious female dominance was observed in patients with early-onset AChR-MG, MuSK-MG, and LRP4-MG, with female: male ratios of 9:1, 4:1, and 2:1, respectively, indicating that sex may affect the pathogenesis of some subtypes (10, 38). The clinical severity is modulated by menstruation, which is abolished by thymectomy, and aggravation occurs during pregnancy and the postpartum period (108, 109). The increased estrogen receptor expression in thymocytes and PBMCs in patients with MG induced by the inflammatory environment suggests that estrogens potentially contribute to the MG autoimmune process by affecting cytokine production and B cell activity (29, 110).

A similar female: male distribution (8–9:1) was also observed in patients with NMO/NMOSD in a new comparative population-based study (24), and the ratio has been shown to reach 23:1 in AQP4-IgG-positive patients during fertile periods (28). Moreover, some researchers have reported a more frequent relapse rate in female patients with NMOSD during pregnancy and the postpartum period and an earlier age of NMOSD onset in patients treated with systemic hormone therapy (111, 112). Based on these findings, gender may affect NMO pathogenesis through female hormones, and genetic or epigenetic factors. Estrogen has been postulated to promote autoreactive B cell development with increasing INF I and B-cell-activating factor (BAFF) generation, to decrease autoreactive B cell apoptosis with upregulation of antiapoptotic molecules, to facilitate antibodies production and affect antibodies glycosylation, and to contribute to the pathogenesis of NMO in females (111).

## Innate Immunity in the Initiation of Autoimmunity

Adaptive immunity plays a major role in both MG and NMO, but requires the innate immune system to initiate pathogenesis (93). However, the exact mechanism by which the immune system initiates autoimmunity in both diseases, particularly NMO, remains largely unknown due the lack of ideal animal models.

In early-onset AChR-MG, the pathogenic link between innate immunity and autoimmunity in the thymus is well recognized (92). Obvious IFN and TLR imprinting has been observed in the hyperplastic thymus, which potentially upregulates the α-AChR expression in epithelial and myoid cells (92). In the context of the genetics of susceptibility and predisposing environmental factors, the aberrant innate response to thymic inflammation may induce AChR sensitization and lead to adaptive immunity (92). In a recent study, an intraperitoneal injection of polyinosinic–polycytidylic acid, a mimic of double-stranded RNA, upregulated TLR3 and IFN-β expression, and stimulated α-AChR overexpression in thymic epithelial cells (TEC), specifically triggering the proliferation of B cells, the generation of anti-AChR antibodies and the presentation of MG-like clinical signs (113). In another study, EBV infection was observed in B cells and plasma cells (PCs) in the thymus of patients with MG (93). Taken together, these findings provide a possible theoretical basis for the mechanism by which innate immunity induces autoimmunity in MG.

Due to the limited data available, researchers have not clearly determined whether the initiation of autoimmunity occurs in the CNS or the periphery in patients with NMO. Levy et al. speculated that astrocyte death induced by an unknown cause might lead to the activation of microglia and the release of inflammatory mediators, thereby disrupting the blood–brain barrier (BBB) and recruiting immunocompetent cells from the periphery. APCs phagocytose cell debris, process the AQP4 antigen, present the linearized determinants to CD4^+^ T cells, and initiate adaptive immunity (114). However, this hypothesis does not explain why AQP4 expressed in peripheral organs does not elicit autoimmunity. In the study by Zamvil et al., *C. perfringens* was overrepresented in the gut microbiome of patients with NMO compared with that of controls and the ABC protein on the bacteria reacted with AQP4 p61–80-specific T cells obtained from patients with NMO and induced Th17 polarization (90, 91). Based on this observation, a molecular mimicry mechanism in the periphery might initiate autoimmunity in patients with NMO (91), a process that must involve the innate immune system. In fact, monocytes derived from the PBMCs of patients with NMO produce more IL-6 for Th17 polarization when stimulated *in vitro* (91).

## Adaptive Immunity

### T Cells

#### T helper 1 (Th1)/Th2/Th17 Cells

T helper 1 cells, Th2 cells, and Th17 cells are important subtypes of CD4^+^ T cells characterized by the different patterns of cytokines they secrete. Th1 cells produce IFN-γ and are responsible for the defense against intracellular pathogens; Th2 cells produce IL-4, IL-5, and IL-13 and are involved in the response to parasitic infections; and Th17 cells produce IL-17 and defend against extracellular pathogens (115). Under pathological conditions, Th1 and Th17 cells are associated with autoimmunity, and Th2 cells are implicated in allergic responses (115).

Patients with MG display increased numbers of IFN-γ or IL-4-expressing cells in PBMCs, suggesting that both Th1 and Th2 cells are involved in MG (29, 116). However, in a recent study, the percentage of Th1 cells among CD4^+^ T cells was higher than the percentage of Th2 cells, and the Th1/Th2 ratio correlated positively with clinical severity in the glucocorticoid-treated group (117). Increased numbers of Th17 cells and serum IL-17 levels were observed in patients with MG complicated with thymoma, and a correlation was observed between the percentage of Th17 cells and the AChR antibody titer (118, 119). In addition, a Th1/Th17/follicular Tfh signature was revealed in an analysis of the transcriptomes of purified thymic T cells obtained from patients with MG, most of whose thymus glands bore germinal centers (GCs) (120). However, in a heterogeneous group of patients with AChR-MG, the serum IL-17 levels were comparable to those of normal controls (121). Increased frequencies of Th1 and Th17 cytokines were detected in patients with MuSK-MG (122), although another study revealed that similar polarization was detected in PBMCs only after stimulation *in vitro* (123).

Some debate exists about the roles of Th1/Th2 cells in NMOSD. Using flow cytometry to analyze T cell subsets in PBMCs, Uzawa et al. observed a higher Th1/Th2 ratio in patients with MS but not in patients with NMOSD (124). In contrast, Shimizu et al. reported a higher Th1/Th2 ratio in patients with NMOSD than in patients with MS (125). However, it is generally accepted that Th17-related cytokine and chemokine levels are frequently elevated in the serum and CSF of patients with NMOSD (126–129). Among these cytokines, IL-6 is secreted by macrophages, dendritic cells, and B cells, induces B cells to synthesize antibodies, and facilitates the differentiation of naïve T cells into Th17 cells (127). Several studies have reported elevated serum and CSF IL-6 levels and a strong correlation between CSF IL-6 levels with clinical signs in patients with NMO (128), and IL-6 receptor blockade results in a decreased relapse rate (130). Levels of granulocyte colony-stimulating factor, which is responsible for the survival, proliferation, and differentiation of neutrophils, and IL-8, which is responsible for neutrophil recruitment, were also increased in the CSF of patients with NMO (127). Taken together, these observations suggest the important roles of Th17 cells in NMO.

#### Treg Cells

CD4^+^CD25^+^FoxP3^+^ Treg cells form a special subgroup of CD4^+^ T cells that are involved in the induction and maintenance of immune homeostasis and tolerance (131). Treg cells can suppress activated T cells and B cells by secreting transforming growth factor-β (TGF-β), IL-10, and IL-35 (131). Defective function of Tregs with reduced FoxP3 expression has been observed in the thymus and PBMCs of patients with AChR-MG (132, 133), but the normal Treg numbers in this study contradict the reduced Treg numbers in PBMCs found in another study (134). It remains unclear whether the Tregs number is abnormal or not in patients with NMO.

#### Tfh Cells

Helper T cells comprise a group of effector T cells that are characterized by the expression of transcription factor B cell lymphoma 6 and the surface marker CD4, C–X–C motif chemokine receptor 5 (CXCR5) and programmed cell death protein 1 (PD-1), which promote B cell maturation and antibody production (135). In our previous study, patients with generalized MG displayed significantly increased numbers of circulating Tfh cells and reduced numbers of follicular Tregs in PBMCs, and the numbers of Tfh cells were strongly correlated with the plasma cell frequency and AChR antibody titers (136). In addition, B cells produced antibodies in an IL-21 signaling-dependent manner when cocultured with Tfh cells (136). As in patients with MG, the Tfh cell frequency was higher in patients with NMOSD than in healthy controls and was higher in relapsing patients than in remitting patients (137). In addition, treatment with methylprednisolone decreased the numbers of Tfh cells in patients with NMOSD (137). Interestingly, in another report, the numbers of circulating memory Tfh cells were increased in patients with NMOSD and were positively correlated with the clinical severity and AQP4 antibody levels (138). Based on these findings, Tfh cells might contribute to the development of MG and NMO through an effect on autoreactive B cells.

### B Cells

#### B Cells, Plasmablasts (PBs), PCs and Memory B Cells (MB)

Both MG and NMOSD are humoral immunity-mediated autoimmune diseases, and B cells play an important role in the pathogenesis of both disorders. PBs and PCs secrete antibodies, and MBs produce proinflammatory cytokines and exacerbate autoimmunity (111). The survival, maturation, and differentiation of B cells is regulated by BAFF (139).

In patients with MG, B cells proliferation is not detected in the peripheral blood, but GCs are observed in the hyperplastic thymus (38), in which B cells encounter the antigen, interact with Tfh, and differentiate into short-lived or long-lived PCs, IgD^−^CD27^−^ B cells (DN), and MBs (140). Higher titers of AChR antibodies are produced by PBs or PCs in the thymus than in peripheral blood cells *in vitro*, but extra-thymic PCs also contribute to serum AChR antibody titers, based on the observation of persistent antibody generation after thymectomy (140). MBs are also involved in MG pathogenesis. In a recent case study of a patient with AChR-MG, relapse occurred after the discontinuation of rituximab and other drugs, with the repopulation of DNs and IgD^−^CD27^+^ MBs (141). The serum BAFF levels in patients with MG were significantly elevated, although the levels were not correlated with the clinical severity (140).

In patients with NMO, the numbers of CD19^int^CD27^high^CD38^high^CD180^−^ B cell PBs are selectively elevated in the peripheral blood and further expanded during relapse; these cells are responsible for the generation of AQP4 antibodies in an IL-6-dependent manner (142). Rituximab was also reported to control clinical activity by reducing the number of CD27^+^ MBs but not by inducing changes in AQP4 antibody levels, which indicates the role of MBs in the development of NMO (143, 144). The serum and CSF BAFF levels were significantly elevated in patients with NMO and the serum BAFF levels were reduced after treatment with rituximab (111).

#### Regulatory B Cells (Breg)

Regulatory B cells comprise a specific group of B cell subsets characterized by production of anti-inflammatory cytokines, such as TGF-β, IL-10, and IL-35, which downregulate excessive immune and inflammatory responses (145). Several studies have observed impaired Bregs in patients with MG. The frequency of CD19^+^CD1d^high^CD5^+^ and CD19^+^CD24^high^CD38^high^ subsets and IL-10-producing B cells (B10) was decreased in patients with MG, and that was correlated with clinical severity (146). In another report, the production of IL-10 and TGF-β1 was lower in patients with MG than in healthy controls (147). Similarly, Quan et al. observed a decreased frequency of CD19^+^CD24^high^CD38^high^ subsets and B10 in patients with NMO compared with those in patients with MS and controls, and the frequency was even lower in patients with AQP4-IgG-positive NMO (148).

## Antibodies

Increasing numbers of antibodies have been discovered in patients with MG, including MuSK antibodies, LRP4 antibodies, agrin antibodies, titin antibodies, potassium voltage-gated channel subfamily A member 4 (K_V_1.4) antibodies, ryanodine receptor antibodies, and others (4). However, AChR antibodies remain the most important antibodies in MG and are present in approximately 80% of patients (4). As the earliest recognized antibody in MG, its pathogenic mechanism has been clarified. (i) Antibodies binding to AChR can accelerate the degradation of AChR by cross-linking the receptors; (ii) AChR is blocked by steric hindrance; and (iii) the complement cascade is activated to form membrane attack complex (MAC) and induces damage to postsynaptic membranes by complement-dependent cytotoxicity (CDC) (149).

Aquaporin-4 antibodies are detected in more than 75% of patients with NMO (11), and MOG and AQP1 antibodies have also been discovered in both AQP4-IgG-positive and -negative patients (13, 23). First, AQP4 antibodies mainly comprising the IgG1 isotypes promote complement cascade activation to form the MAC in the end-feet and lead to cell death by CDC after binding to AQP4 on astrocyte (18). The released inflammatory medium, such as complement protein 3a (C3a) and C5a, together with other cytokines, recruit granulocytes and macrophages, which induce secondary oligodendrocyte damage, demyelination, and neuronal death through antibody-dependent cell-mediated cytotoxicity (ADCC) (150, 151). This mechanism provides an explanation for the typical necrotic lesions observed in the spinal cord, which are characterized by the extensive loss of AQP4 and glial fibrillary acidic protein (GFAP), the perivascular deposition of Igs and activated complement, and the massive infiltration of macrophages and polymorphonuclear leukocytes (39, 152, 153). However, vascular fibrosis and hyalinization in both active and inactive lesions has not been well explained, and a recent finding of glucose-regulated protein 78 (GRP78) autoantibodies targeting endothelial cells in the serum of patients with NMO may provide a new explanation for the vascular involvement and disruption of the BBB (154). Second, an alternative lesion pattern with prominent loss of AQP4 and GFAP but variable absent complement deposition was observed in the area postrema (155). Internalization of AQP4 caused by AQP4-IgG observed *in vitro* was examined in an attempt to decipher the lesion pattern (156), but this was debated because it does not occur *in vivo* (18).

## Autoimmune Comorbidities

Several studies have investigated autoimmune comorbidities in patients with MG. Approximately 15% of patients with MG are also diagnosed with another autoimmune disorder, which most frequently afflicts patients with early-onset AChR-MG (1). Among these disorders, autoimmune thyroid disease (ATD) is the most common in 10% of patients with MG, followed by SLE (1–8%) and RA (4%) (33), and the most common antibodies comprise antithyroid peroxidase antibodies, antithyroglobulin antibodies, antinuclear antibodies, and rheumatoid factor (157). Interestingly, patients with thymoma MG are more susceptible to autoimmune disorders after thymectomy than before surgery, probably due to an altered T cell repertoire (33).

Associations between NMOSD and other autoimmune diseases have also been recognized. Up to 30% of patients with NMOSD are diagnosed with a coexisting autoimmune disease, and 40% of NMOSD patients present other autoantibodies without an obvious accompanying disease (13). The most common diseases, include SLE, SS, MG, ATD, and antiphospholipid syndrome, whereas the most common antibodies comprise anti-extractable nuclear antigens antibodies, anti-SSA and anti-SSB autoantibodies, and rheumatoid factor (13). In most reported cases, the onset of SLE preceded NMOSD by several years, whereas NMOSD symptoms preceded SS by a few years (158).

Regarding the mechanisms of the associated comorbidities in patients with NMOSD, the common genetic and environmental factors have been postulated to facilitate autoimmunity, and the autoimmune comorbidities might partially contribute to the immunopathogenesis of NMOSD (32). Similar mechanisms might also apply to the comorbidities in patients with MG.

Of all the abovementioned coexisting diseases, the co-occurrence of MG and NMOSD in patients arouses much particular interest in researchers, because this is more frequent than expected in the general population (159). In one study of 117 patients with NMOSD, comorbid MG was identified in 2% of patients, and AChR antibodies were detected in 11% of patients (160). In another study of 164 patients with MG, 10–15% of patients had CNS involvement resembling an NMO-like disease, half of whom exhibited AQP4-IgG (161). MG likely has a benign course, but CNS involvement is potentially more severe when accompanied by thymomas (159, 161). AChR antibodies and AQP4 antibodies may precede the onset of the relevant symptoms, and the titers of the two antibodies tend to be negatively correlated (159). In most cases, MG symptoms preceded the onset of NMOSD, and only a few patients developed MG after NMOSD onset (158). Most of these patients had early-onset AChR-MG, and 70% had a history of thymectomy (158). AQP4 is expressed in the thymus, and this may provide a pathogenic basis similar to that of AChR in MG (158). Additionally, the decrease in the number of Tregs following thymectomy may further contribute to NMOSD development (158). AQP4 is also expressed at the NMJ; thus, the degeneration of the postsynaptic membrane induced by AChR antibodies was postulated to initiate AQP4 sensitization in the context of the inflammatory environment in MG, and then mediate the autoimmunity against AQP4 (161).

## Specific Involvement of Different Target Organs

Nicotinic acetylcholine receptors (nAChRs) are expressed in both muscle and brain (34), but MG seldom involves the brain, with the exception of rare reports of cognitive impairment, epilepsy, Parkinson’s disease, MS, and psychological and sleep disorders (35, 161). In addition to the major protective role of the BBB, the differences in structure between muscle and neuronal AChRs consisting of different subunits might also contribute to the brain exemption (34). In fact, antibodies from patients with MG do not bind to nAChRs in the human brain (162). In patients with AChR-MG, extraocular muscle weakness usually precedes generalized muscle weakness, and in patients with ocular MG, extraocular muscle weakness is the sole symptom, along with a lower titer of AChR antibodies (2, 163). Thus, the extraocular muscles are more susceptible to MG and many studies have attempted to explain this specificity. First, extraocular muscles have special physiological features: the neuron innervating extraocular muscles have higher firing frequencies, and the tonic fibers in extraocular muscles depend on intact AChR function, which reduces the endplate safety factor (30). Second, the expression of the complement-regulating proteins CD55 and CD59 is lower in extraocular muscles than in other muscles (164). Third, researchers are still debating whether fetal AChR expressed in extraocular muscles is a target of autoimmunity in MG (165).

The NMO target protein AQP4 is expressed not only in the CNS but also in some peripheral organs, including the kidney, skeletal muscle, stomach, and airways (166); however, the peripheral organs are relatively spared, except for a few reports of myopathy (36), even though the AQP4-IgG titer is higher in the serum than in the CSF (167). Several observations might help to explain this question: (i) AQP4 is higher expressed in CNS than in peripheral organs (168), (ii) a higher ratio of M23/M1 AQP4 isoforms with larger orthogonal arrays of particles (OAPs) of AQP4 in peripheral organs results in a higher capacity to bind to AQP4-IgG and induce CDC (168–171), (iii) the complement regulatory proteins CD46, CD55, and CD59 are expressed in AQP4-expressing cells in peripheral organs but are absent in the astrocytes, as we and others recently reported (36, 37). In addition, larger OAPs in the spinal cord and optic nerve than in the brain may also contribute to the more frequent and severer involvement of the spinal cord and optic nerve in NMO (168). Even in the brain, the typical lesions are distributed in specific location, i.e., the hypothalamic and periventricular areas (172). Higher AQP4 expression in these areas is thought to be responsible for the specific distribution (172). Recently, the involvement of the pia, ependyma, and choroid plexus was observed in 23 autopsy cases of NMO/NMOSD (173). The disruption of the blood–CSF barrier in the choroid plexus was suspected to provide a route for AQP4-IgG to enter the CNS (173), and this may offer another possible explanation for the aforementioned specific distribution in the brain—the areas may be more accessible to the penetration of AQP4-IgG from the CSF and resemble ventriculitis and leptomeningitis in patients with NMO.

## The Panorama of Immunopathogenesis

**Figure 1:**
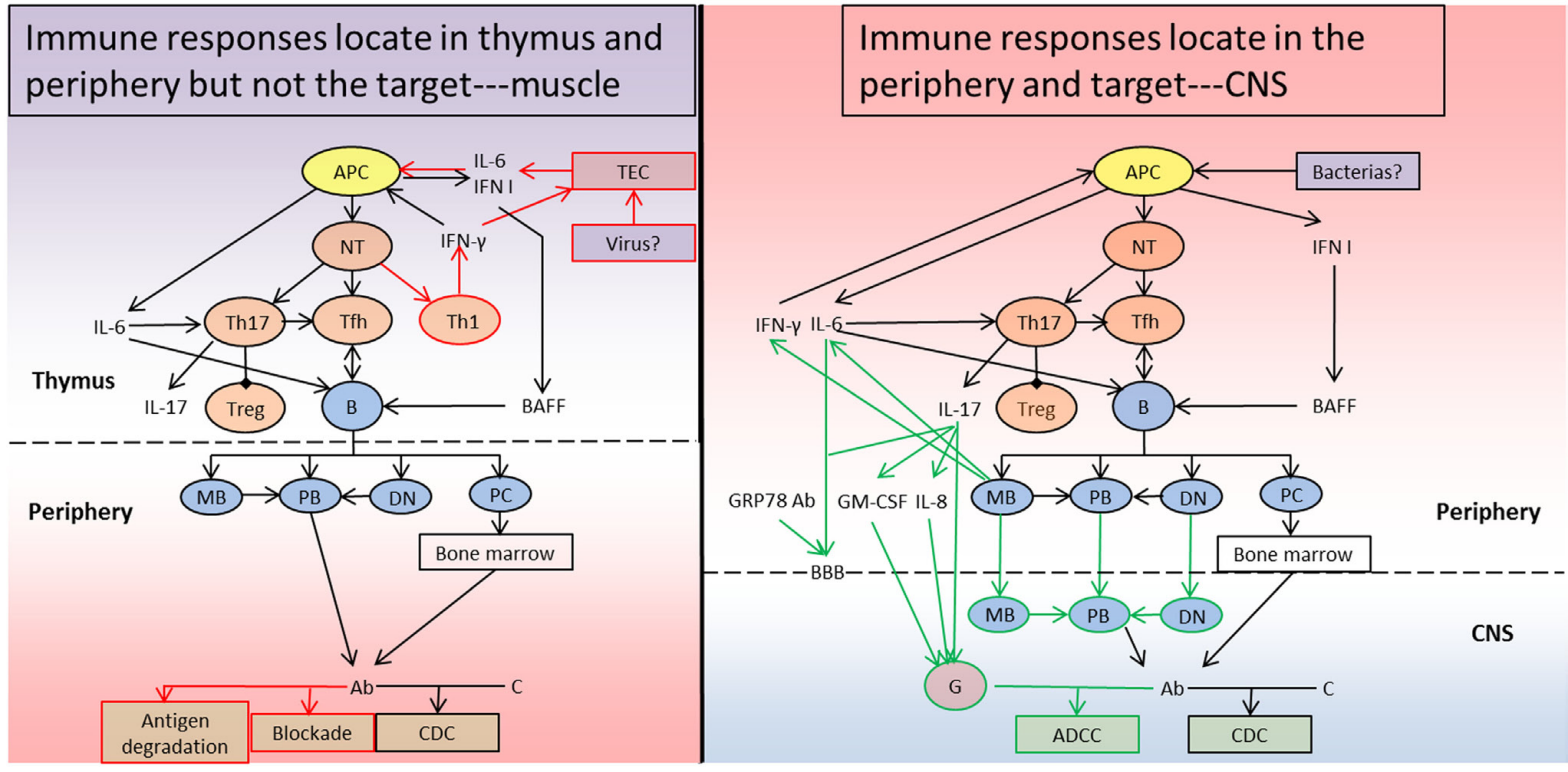
Schematic diagram of the immunopathogenesis of myasthenia gravis (MG) and neuromyelitis optica (NMO). (Left) In the context of susceptible genetic and environmental predisposing factors in MG patients, the TECs secrete IL-6 and IFN I and upregulate acetylcholine receptor (AChR) expression after a triggering event such as viral infections. Together with the effects of the cytokines, APCs phagocytose, process, and present the AChR antigen to naïve T cells and initiate Th1, Th17, and Tfh subsets differentiation. Th1 cells and APCs generate IFN-γ, IFN I, and IL-6 to sustain and amplify the chronic inflammation. Th17 cells produce IL-17 and IL-21 to inhibit Tregs and favor Tfh development. Tfh cells interact with B cells to form germinal centers and promote B cell maturation and antibody production with the help of BAFF and IL-6. MBs, PBs, DNs, and PCs enter into the periphery from the thymus, and MBs and DNs also differentiate into PBs generating Ab, and PCs migrate into the bone marrow to produce Ab. The Ab can destroy the postsynaptic membrane by promoting antigen degradation, blocking functional sites and inducing CDC. (Right) Similar to MG, NMO also develops on the basis of susceptible genetic and environmental predisposing factors. During the priming process (for example, due to infection with particular bacteria), APCs phagocytose the pathogen and present a specific peptide to naïve T cells, which is identical with a particular peptide sequence in the aquaporin-4 (AQP4) protein. Additionally, APCs secrete IFN I to facilitate BAFF generation. The autoreactive naïve T cells then differentiate into Th17 and Tfh subsets. Th17 cells can produce IL-17 and suppress Tregs, and help Tfh development. Tfh cells promote cognate B cells maturation and differentiation into MBs, PBs, DNs, and PCs, together with BAFF. The proinflammatory MBs can further contribute to APC activation, Th17 differentiation, and B cell maturation through IL-6 and IFN-γ. IL-17 and IL-6 or GRP78 Ab can break the BBB and permit MBs, DNs, and PBs to enter into the CNS. The MBs and DNs can also progress to PB to generate Ab, which can target the AQP4 protein in astrocytes together with the antibodies produced by PCs in bone marrow. The Ab attack the astrocytes through CDC, which not only forms membrane attack complex but also generates C5a and C3a recruiting granulocytes in combination with IL-17, IL-8, and GM-CSF. The granulocytes can further aggravate the CNS lesion through ADCC. TEC, thymic epithelial cell; APC, antigen-presenting cell; NT, naïve T cell; Th1, T helper 1 cell; Th2, T helper 2 cell; Th17, T helper 17 cell; Treg, regulatory T cell; Tfh, follicular helper T cell; B, B cell; MB, memory B cell; PB, plasmblast; DN, CD27^−^IgD^−^ double negative B cell; PC, plasma cell; G, granulocyte; IL-6, interleukin 6; IL-8, interleukin 8; IL-17, interleukin 17; IFN I, type I interferon; IFN-γ, interferon γ; BAFF, B cell-activating factor; Ab, antibody; C, complement; GM-CSF, granulocyte-macrophage colony-stimulating factor; GRP78 Ab, glucose-regulated protein 78 antibody; BBB, blood–brain barrier; ADCC, antibody-dependent cell-mediated cytotoxicity; CDC, complement-dependent cytotoxicity; CNS, central nervous system. The red pathway represents the specific immune responses in MG, the green pathway refers to the unique immune responses in NMO, and the black pathway is shared by both disorders. The “periphery” means outside of thymus in MG and outside of CNS in NMO.

In the abovementioned portions, we describe the profiles of the immunopathogenesis of both disorders, particularly the early-onset AChR-MG and AQP4-IgG positive NMO/NMOSD. In general, both diseases are T cell-mediated and B cell-dependent autoimmune channelopathies on the basis of the susceptible gene and predisposing environmental factors. The schematic of immunopathogenesis in MG and NMO is shown in Figure 1, and the comparison between them is summarized in Table 1.

**Table 1:** The comparison of immunopathogenesis between MG and NMO.

	MG	NMO
Susceptible gene	HLA (AH8.1, HLA-B*08:01, HLA-C*07:01, HLA-DQA1, HLA-DQB1, HLA-DQB1*05:02, HLA-DQA1, DQ9, HLA-DRB1*09, DQ*5); non-HLA (CTLA4, TNFRSF11A, ZBTB10, PTPN22, TNIP1, RANKL, CHNRA1, CHRND)	HLA (DPB1*1501, DPB1*0501, DRB1*03); non-HLA (TCR, CD6, TNFRSF1A, CD58, IL-17A, IL-17F, CYP7A1 promoter)

MicroRNA	miR-150 and miR-21 (↑ in sera)	miR-150 and miR-21 (↓in whole blood)

Environmental factors	Vitamin D deficiency, Epstein–Barr virus (EBV)	Vitamin D deficiency, *Clostridium perfringens*, EBV, human T cell leukemia virus type 1 virus

Gender bias	Early-onset AChR-MG, MuSK-MG, and LRP4-MG	AQP4-IgG positive patients

T helper 1 (Th1)/Th2/Th17 cells	Th1/Th2 cells (ND); Th17 cells (↑), serum Th17 cytokines (↑)	Th1/Th2 cells (ND); serum and CSF Th17 cytokines (↑)

Treg cells	FoxP3 expression (↓)	ND

Follicular T cells	Follicular helper T cells (Tfh) (↑); follicular regulatory T cells (↓)	Follicular Tfh (↑)

B cells	B cells (↑ in Thymus); memory B cells (↑ during relapse); serum BAFF levels (↑); Bregs (↓), IL-10 and TGF-β1 (↓)	Plasmablasts (↑); memory B cells (↑ during relapse); serum and CSF BAFF levels (↑); Bregs (↓)

Antibodies	AChR antibodies (80%), MuSK antibodies (1–10%), LRP4 antibodies (1–5%), agrin antibodies (minority), titin antibodies (20–30% of AChR-MG), potassium voltage-gated channel subfamily A member 4 antibodies (10–20%), ryanodine receptor antibodies (70% of MG with thymoma and 14% of late-onset AChR-MG)	AQP4 antibodies (>75%), myelin oligodendrocyte glycoprotein antibodies (5–10%), AQP1 antibodies (26%), glucose-regulated protein 78 antibodies

Autoimmune comorbidities	ATD (10%), SLE (1–8%), RA (4%); TPO antibodies (36%), TG antibodies (23%), ANA (23%), rheumatoid factor (8%), AQP4 antibodies (5–7%)	ATD (17%), SLE (2.0%), SS (2.0%), MG (2%), RA (1.3%); ANA (43%), ENA (15%), SS-A (10%), SS-B (3%); and rheumatoid factor (5%), AChR antibodies (11%)

Inflammatory infiltration	B cells and macrophages in the thymus, absent in muscle	Polymorphonuclear leukocytes and macrophages in CNS

*MG, myasthenia gravis; NMO, neuromyelitis optica; HLA, human leukocyte antigen; CTLA4, cytotoxic T lymphocyte-associated protein 4; TNFRSF, tumor necrosis factor receptor superfamily; ZBTB10, zinc finger and BTB domain-containing 10; PTPN22, protein tyrosine phosphatase nonreceptor type 22; TNIP1, tumor necrosis factor alpha-induced protein 3-interacting protein 1; RANKL, receptor activator of nuclear factor κB ligand; TCR, T cell receptor; CD, cluster of differentiation; IL, interleukin; AChR, acetylcholine receptor; MuSK, muscle-specific kinase; LRP4, lipoprotein receptor-related protein 4; AQP4, aquaporin-4; Th, T helper; Treg, regulatory T; FoxP3, forkhead box P3; ND, not determined; BAFF, B-cell-activating factor; Bregs, regulatory B cells; TGF, transforming growth factor; CSF, cerebrospinal fluid; CNS, central nervous system; ENA, extractable nuclear antigen; ANA, antinuclear antibody; ATD, autoimmune thyroid disease; SLE, systemic lupus erythematosus; RA, rheumatoid arthritis*.

After triggering events such as viral infections in patients with MG, the TEC secrete IL-6 and IFN I and upregulate α-AChR expression. The APCs then phagocytose, process, and present a linear peptide of the AChR protein to naïve T cells, thus initiating Th1, Th17, and Tfh subset differentiation (29, 92). Th1 cells and APCs generate IFN-γ, IFN I, and IL-6 to sustain and amplify the chronic inflammation (29, 38). Th17 cells produce IL-17 and IL-21 to inhibit Tregs and favor Tfh development (29, 120). Tfh cells interact with B cells to form GCs and promote B cell maturation and AChR antibody production with the help of BAFF and IL-6 (120, 140). MBs, PBs, DNs, and PCs enter into the periphery from the thymus, MBs, and DNs then also differentiate into PBs to generate antibodies, and PCs migrate into the bone marrow to persistently produce antibodies (140, 141). The antibodies destroy the AChR channel on the postsynaptic membrane by promoting antigen degradation, blocking functional sites, and inducing CDC (149).

Similar to MG, during the priming process in patients with NMO (for example, with infections of some bacterias), APCs phagocytose the pathogen and present a specific peptide to naïve T cells; this peptide is identical to a peptide sequence in the AQP4 protein (91). In addtion, APCs secrete IFN I to facilitate BAFF generation (111). The autoreactive naïve T cells then differentiate into Th17 and Tfh subsets. Th17 cells can produce IL-17, suppress Tregs, and help Tfh development (126, 127). Together with BAFF, Tfh cells can promote cognate B cell maturation and differentiation into MBs, PBs, DNs, and PCs (111, 137, 174, 175). The proinflammatory MBs can further contribute to APC activation, Th17 differentiation, and B cell maturation through IL-6 and IFN-γ (111, 174). IL-17 and IL-6 or GRP78 antibodies can break the BBB and permit MBs, DNs, and PBs to enter the CNS (126, 127). The MBs and DNs can also progress to PBs to generate antibodies, which can target the AQP4 protein in astrocytes, together with the antibodies produced by PCs in bone marrow (175). The antibodies attack the astrocytes through CDC, which not only forms MAC but also generates C5a and C3a recruiting granulocytes in combination with IL-17, IL-8, and GM-CSF (111, 127, 150). The granulocytes can further aggravate the CNS lesions through ADCC (150, 176, 177).

At last, it is noteworthy that it is still in debate about the classification and the role of the DNs (178, 179). The DNs are very likely identical to the atypical memory B cells, tissue-like memory B cells, or age associated B cells (179). The conclusions are conflicting if the DNs play an immune boosting or tolerant role in autoimmune disease, such as RA or SLE, and chronic infection such as human immunodeficiency virus, malaria, or hepatitis C virus (179), which suggest the exact roles of the DNs in the development of MG and NMO should be further studied. Besides, in addition to promoting the survival and differentiation of autoreactive B cells in mature stage (139), BAFF can also facilitate the proliferation and antibody-secretion of immature-transitional B cells (180), indirectly promote the expansion of Th17 cells in RA and directly regulate the accumulation and cytokine-secretion of Tfh cells in SLE (181, 182), which is likely to be also involved in the pathogenesis of MG and NMO and deserves to be investigated.

## Conclusion

Both of MG and NMO are developed on the basis of susceptible gene and environmental predisposing factors, which initiate the innate immunity and activate the adaptive immunity (29, 114). The autoreactive T cells cooperate with cognate B cells to generate effector and memory lymphocytes (29, 31). The autoantibodies attack NMJ together with complement in MG (29). And the autoantibodies together with complement and inflammatory cells destroy CNS after breaking BBB in NMO (114). In this review, we summarize the similarities and discrepancies between MG and NMO, including the genetics, environmental factors, gender bias, innate immunity, adaptive immunity, autoimmune comorbidities, and specific involvement. This review will help to improve our understanding of the pathogenesis, promote the mutual exchange of information in future progress regarding immune mechanisms, and facilitate the two-way communication between MG and NMO regarding new therapeutic strategies in future clinical trials. In the future, the genetic and epigenetic analysis of these patients, especially the monozygotic twins, can further unravel the pathogenic basis of both diseases; the dynamic detection of the immune cells and molecules in these patients, especially those with monoclonal antibodies therapy, will clearly decipher the pathogenic process; the development of appropriate animal models, especially in NMO, will pave the way for the drug development.

## Author Contributions

ZW and YY wrote and approved the final version of this manuscript.

## Conflict of Interest Statement

The authors declare that the research was conducted in the absence of any commercial or financial relationships that could be construed as a potential conflict of interest.
